# Contribution of ***α***- and ***β***-Adrenergic Mechanisms to the Development of Pulmonary Edema

**DOI:** 10.6064/2012/829504

**Published:** 2012-08-07

**Authors:** Beate Rassler

**Affiliations:** Carl Ludwig Institute of Physiology, University of Leipzig, Liebigstraße 27, 04103 Leipzig, Germany

## Abstract

Endogenous or exogenous catecholamines can induce pulmonary edema (PE). This may occur in human pathologic conditions such as in pheochromocytoma or in neurogenic pulmonary edema (NPE) but can also be provoked after experimental administration of adrenergic agonists. PE can result from stimulation with different types of adrenergic stimulation. With *α*-adrenergic treatment, it develops more rapidly, is more severe with abundant protein-rich fluid in the alveolar space, and is accompanied by strong generalized inflammation in the lung. Similar detrimental effects of *α*-adrenergic stimulation have repeatedly been described and are considered to play a pivotal role in NPE or in PE in patients with pheochromocytoma. Although *β*-adrenergic agonists have often been reported to prevent or attenuate PE by enhancing alveolar fluid clearance, PE may also be induced by *β*-adrenergic treatment as can be observed in tocolysis. In experimental models, infusion of *β*-adrenergic agonists induces less severe PE than *α*-adrenergic stimulation. The present paper addresses the current understanding of the possible contribution of *α*- and *β*-adrenergic pathways to the development of PE.

## 1. Introduction 

Strong sympathetic activation or highly elevated plasma concentrations of epinephrine or norepinephrine (NE) play a pivotal role in several types of pulmonary edema (PE) such as in patients with pheochromocytoma or in neurogenic pulmonary edema (NPE). PE may develop as a consequence of therapeutic administration of catecholamines (CAs) or sympathomimetic drugs, for example, for surgical interventions, for tocolysis, or in patients with cardiocirculatory insufficiency.

We established a model of continuous intravenous (i.v.) infusion of CAs in rats that allowed investigation of the time course of edema formation. Moreover, we could compare the effects of NE with those of selective *α*- or *β*-adrenergic agonists as well as of combinations of NE with *α*- or *β*-adrenergic blockers. The present paper outlines the possible contribution of *α*- and *β*-adrenergic pathways to the development of pulmonary edema in clinical and experimental conditions. 

## 2. Catecholamine-Associated Pulmonary Edema in Clinical Conditions

### 2.1. PE in Patients with Pheochromocytoma

Patients with pheochromocytoma can develop PE as a rare life-threatening complication. In some cases, it even can occur as the first manifestation of this CA-releasing tumor [1–4]. It mostly presents as cardiogenic PE [1, 5], but in rare cases noncardiogenic edema has been described [3, 4, 6–10]. Cardiogenic PE is associated with left ventricular (LV) dysfunction, while noncardiogenic edema presents with normal LV function and without signs of cardiopathy. Several mechanisms are proposed for pathogenesis of noncardiogenic PE which all can be induced by elevated CA levels or sympathetic discharge, mainly by predominant *α*-adrenergic stimulation: (1) vasoconstriction, especially postcapillary venoconstriction in the lung resulting in a rise in pulmonary-capillary hydrostatic pressure, (2) elevated pulmonary blood volume, (3) increased alveolocapillary permeability, and (4) inflammation with neutrophil accumulation in the lung [2, 6, 7, 9]. The latter may be caused by interleukin-6 (IL-6) overproduction that is thought to be a consequence of high CA levels [11, 12]. 

In a pheochromocytoma patient who had been successfully treated from noncardiogenic pulmonary edema induced by massive endogenous CA release, exogenous NE administration provoked reformation of edema with similar characteristics. Application of *α*-adrenergic blockers resulted in prompt and sustained relief of all symptoms, thus confirming the crucial role of *α*-adrenergic mechanisms in the development of this type of PE [6]. *α*-Adrenergic blockers have also been applied in the treatment of pheochromocytoma multisystem crisis [13]. Pathogenesis of PE in patients with pheochromocytoma is thought to be similar to NPE, which is believed to result from massive *α*-adrenergic stimulation caused by a discharge of sympathetic activity [6–8].

### 2.2. Neurogenic PE

NPE may occur as a consequence of a neurological insult due to severe head injury, cerebral hemorrhage, generalized seizures, and so forth. Usually, it impresses by a very sudden onset—even just minutes after the precipitating event—as has been reported from soldiers after bullet head injuries during the Vietnam War [14].

NPE is characterized by interstitial and alveolar edema together with intra-alveolar hemorrhage [15]. Strong sympathetic activation following a sudden increase in intracranial pressure or a brain injury is considered to be the initial step. In an experimental study in rats, massive pulmonary hemorrhagic edema could be produced either by cerebral compression or by epinephrine injection [16]. The released CA surge causes hemodynamic changes with generalized vasoconstriction and a rapid increase in blood volume in the pulmonary vasculature. Vasoconstriction also concerns pulmonary circulation, especially pulmonary veins [17], thus leading to pulmonary congestion and increased pulmonary capillary hydrostatic pressure [18]. Moreover, vasoconstriction causes systemic hypertension, which in turn via baroreflex activation induces bradycardia. Bradycardia accompanying high systemic blood pressure has been recognized as another important factor in formation of NPE. This may be even more important than the elevated pulmonary blood volume as it leads to reduced cardiac output and further elevation of pulmonary venous pressure. Prevention of this baroreflex-induced bradycardia, for example, by early i.v. administration of atropine, can avoid the development of NPE [19]. These findings emphasizing vasoconstriction as the primary hemodynamic factor in NPE formation demonstrate the crucial role of the sympathetic nervous system in this condition. In an experimental study with balloon compression injury of the spinal cord in rats, NPE was completely prevented by inhibition of the sympathetic system with pentolinium administered prior to balloon compression [20]. Although NPE is traditionally considered to be a “noncardiogenic edema,” there is evidence that cardiac dysfunction due to direct myocardial injury may be involved in NPE formation and that CAs induce this myocyte injury [21, 22].

As NPE is characterized by severe proteinaceous alveolar edema, increased permeability of pulmonary vascular walls is generally assumed to be involved in the development of NPE. However, there are findings to the contrary [23]. The elevated capillary pressure may cause disruption of the alveolocapillary barrier and, consequently, intra-alveolar hemorrhage and accumulation of protein-rich fluid [15, 24–26]. An increase in lung microvascular permeability usually occurs when the transmural pressure exceeds 40 mmHg [18]. Additionally, inflammatory mechanisms due to release of brain cytokines and chemokines are considered to contribute to the increase in pulmonary capillary permeability [18, 27, 28]. Stimulation of cytokine expression and inflammation in the lungs may also ensue from the massive sympathetic discharge caused by the cerebral insult [18, 29]. 

*α*-adrenergic mechanisms play a pivotal role in the formation of NPE [18, 30]. Besides vasoconstriction, *α*-adrenergic agonists induce the release of inflammatory mediators and promote an increase in vascular permeability [29]. *α*-adrenergic antagonists are able to prevent the formation of NPE in experimental NPE models [17, 26, 31, 32] and in NPE patients [33, 34], while *β*-blockers failed to prevent NPE formation [35]. Blocking of *α*-adrenoceptors attenuates constriction of pulmonary veins following head trauma in rats [36]. Moreover, it reduces the severity of inflammatory reactions and restores the normal fluid transport capacity in the lung [37]. Recommendations for symptomatic treatment of NPE include *α*-blockers and corticoids, while *β*-blockers should be avoided [18]. 

## 3. Pulmonary Edema Induced by Experimental NE Administration 

With our experimental model, we investigated the effects of NE infusion (1 mg·kg^−1^·h^−1^) in rats over time intervals up to 72 h. This NE dose has previously been shown to induce LV hypertrophy in rats [38] and, hence, was considered to produce a similar NE activity as can be found in patients developing cardiac hypertrophy. We observed a twofold increase in right ventricular systolic pressure (RVSP) within several minutes. First signs of interstitial PE occurred after less than one hour of infusion. Total peripheral resistance (TPR) increased gradually and was significantly elevated after 8 h of infusion. At that time, PE became more pronounced and—as a mechanism of compensation—pleural effusion emerged. TPR was in significant positive correlation with the volume of pleural fluid (PF) indicating a crucial role of hemodynamic changes for development of PE. This was confirmed by treatment with the *α*-adrenergic blocker prazosin that prevented both the increase in TPR and the occurrence of pleural effusion [39, 40]. Our results are in accordance with previous animal studies using single high-dose injections of NE or adrenaline. These treatments provoked pulmonary bleeding and edema resulting from generalized systemic vasoconstriction and elevated blood volume in the pulmonary vascular bed [32, 41].

NE infusion also induced pulmonary inflammation that developed in parallel with PE. Inflammation was reflected by enhanced expression of proinflammatory cytokines such as IL-6, IL-1*α*, and IL-1*β*, and by lung histology. After 8 h of infusion, IL-6 protein concentration in serum was increased to the 6-fold of control. Likewise, elevated IL-6 concentrations were found in PF and bronchoalveolar lavage fluid (BALF). After 12 h, IL-6 protein began to increase in lung tissue and reached significance after 48 h. Lung histology showed strong interstitial and mild alveolar edema as well as moderate inflammation after 24 h of NE infusion. PE gradually disappeared over the next 2 days, while inflammation was even more pronounced after 72 h [42]. 

NE infusion has been shown to induce mRNA expression of IL-6, its transcriptional factors C/EBP-*β* and C/EBP-*δ*, and its receptors in the heart [43]. Moreover, NE also increased IL-1*β* mRNA expression, and this effect was prevented by combined *α*- and *β*-blockade [44]. Pulmonary inflammation can result from strong sympathetic activation. This has been observed in stress situations such as hemorrhage [37, 45, 46] or exercise at high altitude [47, 48]. The authors reported elevated concentrations of proinflammatory cytokines such as IL-1*β*, IL-6, and tumor necrosis factor (TNF)-*α* as well as increased number and function of inflammatory cells in the lung and in BALF. In patients with pheochromocytoma, systemic inflammation associated with overproduction of IL-6 may occur. This is thought to be a consequence of the high levels of circulating NE [11, 12]. 

Inflammatory processes may induce or deteriorate PE by damaging the alveolar-capillary barrier, thus causing extravasation of proteinaceous fluid and flooding of the alveoli. The involvement of inflammation has been described for various types of PE, even for those types that are considered to be initiated by hemodynamic changes in pulmonary circulation such as NPE or high-altitude pulmonary edema (HAPE) [27, 28, 47, 49]. In these PE types, inflammation can maintain and even aggravate fluid and protein shifts.

**Figure 1:**
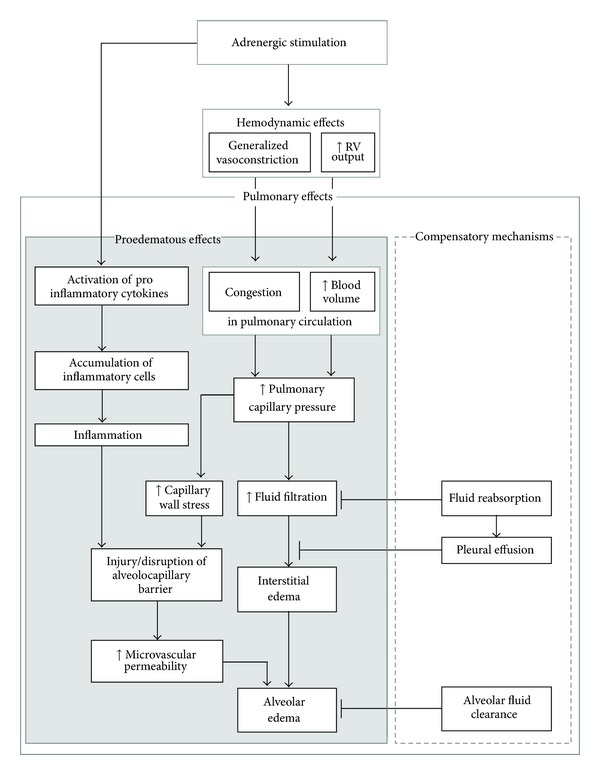
Pathogenic mechanisms of the contribution of adrenergic stimulation to the development of pulmonary edema. Pulmonary effects promoting development of edema are presented on the left side (grey box); protective mechanisms are depicted on the right side (white box). Hemodynamic effects (generalized vasoconstriction and increase in the RV output) result in blood overfilling and congestion in pulmonary circulation and consequently, in elevated pulmonary capillary pressure. This is the primary factor in the development of edema. High microvascular pressure causes capillary wall stress and may lead to disruption of the alveolocapillary barrier. Adrenergic stimulation also promotes proinflammatory processes. The resulting inflammation can deteriorate edema by further increasing capillary permeability. On the right-hand side, antiedematous mechanisms of the lung are shown. Reabsorption processes counteract fluid filtration. Excess fluid can be drained from the interstitium into the pleural space, thus forming pleural effusion. Alveolar fluid clearance eliminates fluid from the air space, thus preventing development of alveolar edema. RV: right ventricular, ↑: increase.

In accordance with these findings, we have interpreted the findings of our experimental model as follows: hemodynamic changes, that is, increase in TPR due to generalized vasoconstriction and elevated RVSP, may induce overfilling and congestion in the pulmonary circulation. As a consequence, pulmonary capillary pressure and hence fluid filtration will increase. The elevated capillary pressure exerts stress to the capillary walls, and the superimposing inflammation may then cause disruption of the alveolar-capillary barrier. If these processes develop slowly, compensatory mechanisms such as alveolar fluid clearance (AFC) and filtration into the pleural space may prevent or attenuate edema formation or, at least, alveolar flooding. While after a massive head injury, NPE can develop within a very short interval of time (e.g., from seconds to a few minutes [14]), our NE infusion model induced a protracted development of edema and, hence, allowed partial compensation by formation of pleural effusion (see Figure 1).

## 4. Responses to *α*-Adrenergic Stimulation 

In our rat model, we also investigated the effects of selective *α*- or *β*-adrenergic stimulation. For *α*-adrenergic treatment, we used either the *α*-adrenergic agonist phenylephrine (PHE, 0.5 mg·kg^*‒*1^·h^*‒*1^) or a combination with NE (0.1 mg·kg^*‒*1^·h^*‒*1^) and the *β*-blocker propranolol (1.0 mg·kg^*‒*1^·h^*‒*1^). With both types of infusion, edema developed more rapidly and was more severe than with NE alone. PE already occurred after less than 1 h of infusion and peaked after 8 h presenting as severe protein-rich alveolar edema [40, 50]. These animals, however, had only small amounts of PF. The severity of the edema depended on the dose of PHE—with a dose of 3 mg·kg^−1^·h^−1^, severe protein-rich edema with hyaline membranes was present after 6 h of infusion. Lung histology strongly resembled that of human acute respiratory distress syndrome (ARDS). In fact, several animals died from edema within the first 24 h of infusion. With the lower dose of 0.5 mg·kg^−1^·h^−1^, no premature deaths were observed [50]. 

These findings are in accordance with those of an earlier study on *α*-adrenergic effects in anesthetized and bivagotomized rats. This study showed that high doses of epinephrine or PHE induced protein-rich alveolar and interstitial edema that rapidly became lethal. Both PE formation and subsequent death could be prevented with *α*_1_-adrenergic blockade, while the selective *α*_2_-adrenoceptor antagonist yohimbine was ineffective [51]. Strong arterial hypertension and increased permeability of pulmonary capillaries due to damage of the capillary walls are crucial pathogenic mechanisms of *α*_1_-adrenergic stimulation in formation of pulmonary edema [51, 52]. 

With *α*-adrenergic treatment, we observed an earlier increase in TPR than with NE infusion, but in contrast, heart rate remained at control level [50]. RVSP only increased with the highest dose of PHE. This may account for the more severe degree of edema in those animals. But even without elevated RVSP, generalized vasoconstriction as reflected by the increased TPR and low cardiac output led to congestion in the pulmonary vascular bed. A similar mechanism with vasoconstriction, increased LV afterload, and decreased cardiac output has been proposed for development of PE after topical or submucosal application of PHE or epinephrine. Some of the patients reported in these studies were then treated with *β*-blockers, but their cardiopulmonary function deteriorated due to reduced contractility and inability to increase heart rate [53, 54]. 

 In addition to these hemodynamic effects, we observed signs of inflammation after infusion with PHE or with NE plus propranolol. They were similar or even more pronounced than with NE infusion. mRNA expression of proinflammatory cytokines such as IL-1*α*, IL-1*β*, and IL-6 increased in lung tissue to 25–60-fold of control values after 45 min. Histology showed extensive inflammation after 24 h that was followed by an increased number of neutrophils in BAL [40, 50]. 

Proinflammatory CA effects are predominantly mediated via *α*_1_-adrenergic mechanisms. In a mouse model of endotoxemia induced by lipopolysaccharide (LPS), administration of the *α*_1_-adrenergic agonist PHE significantly increased mRNA expression of proinflammatory cytokines such as IL-1*β* and TNF-*α* by lung neutrophils, while *α*_2_-adrenergic stimulation prevented endotoxin-induced increases in lung neutrophil cytokines [55]. In a similar rat model, both *α*-adrenergic stimulation and *β*-adrenergic blockade counteracted anti-inflammatory isoflurane effects [56, 57]. Correspondingly, *α*-adrenergic blockade with phentolamine or prazosin attenuated the increase of proinflammatory cytokines in lung tissue and in plasma [37, 48].

**Figure 2:**
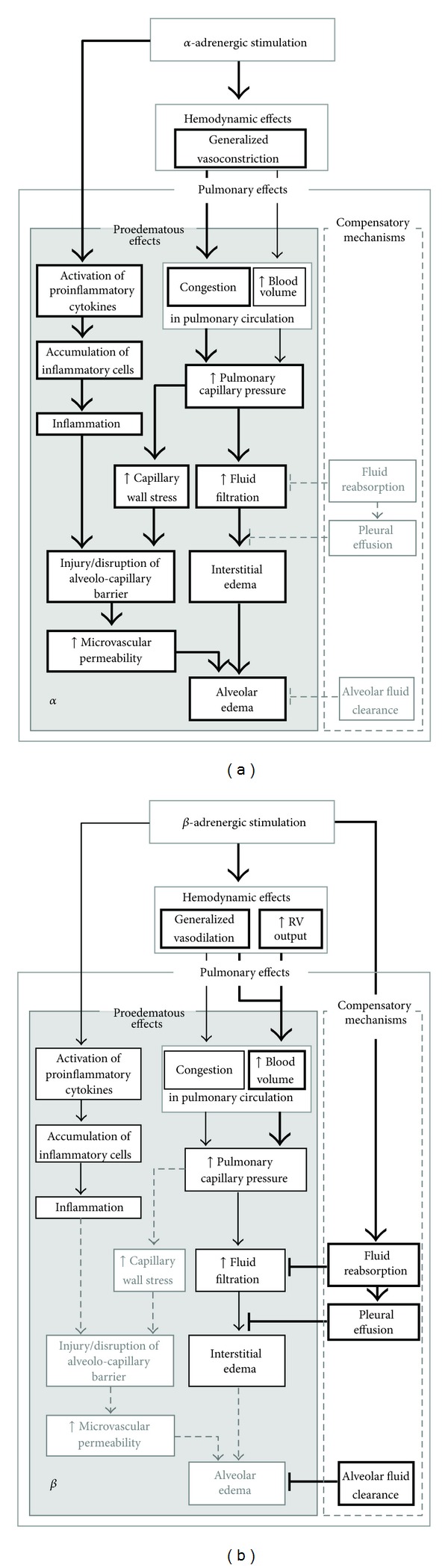
(a): Contribution of *α*-adrenergic stimulation to the development of pulmonary edema. Treatment with *α*-adrenergic agonists promotes all proedematous effects of adrenoceptor stimulation, particularly pulmonary microvascular congestion. Elevated capillary pressure and capillary wall stress increase fluid filtration. Microvascular permeability can increase due to capillary wall stress and inflammation. This may result in alveolar edema. Protective mechanisms are not advanced by *α*-adrenergic mechanisms. (b): Contribution of *β*-adrenergic stimulation to the development of pulmonary edema. Vasodilation mediated by *β*-adrenergic stimulation may cause blood overfilling in the pulmonary circulation and, consequently, increase pulmonary capillary pressure. This is usually less pronounced than with *α*-adrenergic stimulation and is not associated with increased capillary wall stress. Although *β*-adrenergic agonists exert anti-inflammatory effects, prolonged stimulation may induce focal inflammation. In general, with *β*-adrenergic stimulation edema develops slowly allowing protective mechanisms such as filtration into the pleural space to be more effective. Moreover, compensatory mechanisms such as alveolar fluid clearance are enhanced, thus preventing flooding of the alveoli. Bold arrows and boxes depict the main effects of the respective treatment; thin arrows and boxes characterize slight or less pronounced effects; dashed arrows and boxes with light-grey frames and types mark processes that are not affected or inhibited by this type of stimulation. RV: right ventricular, ↑: increase.

These data—in accordance with numerous reports from patients with NPE or with pheochromocytoma—clearly indicate that *α*-adrenergic mechanisms, and particularly stimulation of *α*_1_-adrenoceptors, can induce PE by hemodynamic and proinflammatory effects. In this pathogenesis, the primary change is an increase in pulmonary capillary pressure due to generalized vasoconstriction including constriction of pulmonary veins and a massive blood shift into the pulmonary circulation. Elevated pulmonary vascular pressures produce increases in transcapillary filtration that are more than proportional to the pressure increments. Above a pressure threshold of about 40 mmHg, there is usually an increase in lung microvascular permeability, aggravating the state of PE [18]. In parallel, activation of proinflammatory cytokines and accumulation of inflammatory cells such as neutrophils and lymphocytes occurs in the lung. Inflammation additionally enhances microvascular permeability and thus can intensify and maintain PE (see Figure 2(a)).

## 5. Effects of *β*-Adrenergic Stimulation 

*β*-adrenergic stimulation may also induce pulmonary edema and inflammation as we observed in our experiments after infusion with either isoproterenol (ISO, 0.024 mg·kg^−1^·h^−1^) or NE (0.1 mg·kg^−1^·h^−1^) plus the *α*-adrenergic blocker prazosin (0.1 mg·kg^−1^·h^−1^).

PE was less severe than with *α*-adrenergic treatment. It developed slowly to a mild degree and was always confined to the interstitium. In contrast to *α*-adrenergic stimulation, it was accompanied by large amounts of PF. *β*-adrenergic stimulation significantly increased RVSP but reduced TPR to about 60% of control values. IL-6 concentration in serum, PF, and BALF remained at control level. On the other hand, we observed a transient increase in mRNA expression of IL-1*α*, IL-1*β*, and IL-6 in lung tissue after less than 1 h of infusion. Lung histology showed peribronchial inflammation after 24 h that became even stronger during the following two days and was not significantly less than with *α*-adrenergic infusion after 2-3 days [40, 50].

The much lower degree of pulmonary edema after *β*-adrenergic compared to *α*-adrenergic stimulation corresponds well with numerous reports from clinical and experimental observations. Both in patients with pheochromocytoma and with NPE, edema formation was mainly associated with *α*-adrenoceptor stimulation, and consequently, treatment with *α*-adrenergic antagonists was effective with respect to edema resolution [6, 13, 33, 34]. Several cases of PE following intraoperative topical application of PHE have been reported [58, 59]. Such complications, especially an intraoperative death of a child, finally led to the establishment of guidelines for the use of PHE in the operating room [60]. Correspondingly, PE may also be provoked by therapeutic application of *β*-blockers as has been described in a patient with pheochromocytoma after oral propranolol therapy. The authors assumed that PE may have developed as a consequence of unopposed *α*-adrenergic effects and sudden elevation of cardiac afterload due to *β*_1_- and *β*_2_-blockade [61].

Several antiedematous effects of *β*-agonists have been described. Firstly, stimulation of *β*-adrenergic receptors (ARs) increases AFC, which is an important mechanism of edema prevention and resolution (for a review, see [62]). Most of the pulmonary adrenoceptors are *β*_2_-ARs which, via cAMP-dependent and -independent pathways, regulate some key proteins of the alveolar epithelial ion and fluid transport such as epithelial sodium and chloride channels and Na^+^-K^+^-ATPase [63–68]. Moreover, stimulation of *β*_2_-AR with terbutaline can induce hyperplasia of alveolar type-II epithelial cells, which is associated with a greater AFC [69].

Secondly, *β*-agonists, particularly *β*_2_-AR agonists, may exert anti-inflammatory effects. They have been shown to counteract inflammatory stimuli both in systemic and pulmonary circulation. Treatment with *β*-AR agonists such as ISO, terbutaline, and others attenuated LPS-induced TNF-*α* secretion in various cells and tissues including lung tissue [70–73]. In human venous blood samples, epinephrine as well as the *β*-adrenergic agonist isoprenaline inhibited LPS-induced production of TNF-*α*, IL-1*β*, and IL-6 [74, 75]. *In-vivo* studies on experimental acute lung injury (ALI) in animals and in humans also confirmed the anti-inflammatory effects of *β*_2_-adrenergic agonists including significant suppression of proinflammatory mediators and cytokines such as IL-6 and TNF-*α*, induction of the anti-inflammatory cytokine IL-10, and reduced neutrophil infiltration in the lung [73, 76–78]. Correspondingly, the *β*-adrenergic blocker propranolol has been shown to counteract the anti-inflammatory effects of volatile anesthetics in a rat endotoxemia model [57]. 

Thirdly, *β*-adrenergic stimulation can decrease the permeability of the alveolar-capillary barrier. In systemic vascular beds, they have been shown to counteract the increase in vascular permeability induced by histamine or bradykinin [26]. ISO infusion in isolated rat lungs significantly attenuated increases in the microvascular permeability induced by high pulmonary venous pressure [79]. Similar protective effects of *β*-AR agonists with reduction of endothelial permeability have been demonstrated both in animal models of lung injury and in patients with ARDS [80–83]. Both in ARDS patients and in cultured pulmonary epithelial cells, salbutamol reduced alveolar-capillary permeability by stimulating epithelial wound repair [84]. Increased intracellular cAMP concentration is an important mediator of decreasing permeability as it reduces proinflammatory responses by macrophages and neutrophils. It also stabilizes the cytoskeleton by regulating myosin light chain phosphorylation in endothelial cells [85, 86]. 

All these beneficial effects of *β*-adrenergic agonists initiated research into therapeutic use of *β*-AR agonists against pulmonary edema. There were many promising findings in the treatment and prevention of HAPE, ALI/ARDS, and other types of PE [84, 87–90]. However, more recent randomized placebo-controlled clinical trials failed to prove a significant benefit of treatment with *β*-AR agonists with respect to clinical outcome or mortality in patients with ALI or ARDS. Hence, routine use of aerosolized or intravenous *β*_2_-agonist therapy in ventilated patients cannot be recommended [91, 92]. 

Our experimental model has shown that infusion of *β*-adrenergic substances may induce pulmonary edema and inflammation; however, this injury was less severe than with *α*-adrenergic stimulation [40, 50]. Obstetricians have repeatedly reported cases of acute PE after tocolytic treatment with *β*-AR agonists such as ritodrine or salbutamol [93–97]. However, PE is unusual in nonpregnant patients receiving *β*-adrenergic therapy, for example, antiasthmatic treatment. Several conditions may account for this complication in pregnant women such as increased blood volume, increased cardiac output, hemodilution with reduction of oncotic plasma pressure due to activation of the renin-angiotensin-aldosterone system, iatrogenic fluid overload, increased pulmonary capillary permeability, preexisting cardiovascular disease, and others [93, 98–100]. *β*-AR stimulation can amplify some of these factors, for example, cardiac output and activity of the renin-angiotensin-aldosterone system. Besides our study [40, 50], there are other experimental and clinical reports on edema formation due to *β*-adrenergic stimulation. Rona and coworkers [101] demonstrated the development of PE in a dose-dependent manner with ISO infusion in rats. In a pheochromocytoma patient with a noncardiogenic PE, persistent postural hypotension and tachycardia suggested a predominant *β*-adrenergic hyperactivity [7]. 

*β*-adrenergic stimulation causes vasodilation, increased cardiac output, and elevated central blood volume. Similar hemodynamic features are thought to be involved in the formation of HAPE. Sympathetic activation with elevated cardiac output and vasodilation in combination with inhomogeneous hypoxic vasoconstriction and subsequent regional overperfusion in the lungs may increase pulmonary arterial and capillary pressure. This is considered to be the initial cause of HAPE [102–105]. From the hemodynamic effects of *β*-adrenergic stimulation in our study, especially from the increased cardiac output that reached more than twice the value of NE infusion [40], we would expect pulmonary blood overfilling and, consequently, increased fluid filtration in these animals. 

There are numerous studies reporting anti-inflammatory effects of *β*-adrenergic stimulation. However, some findings indicated that *β*-adrenergic mechanisms may also be involved in proinflammatory reactions. Several studies have demonstrated that prolonged use and high doses of *β*-AR agonists for asthma treatment are associated with increased generation of proinflammatory mediators. This adverse effect is based on the blockade of anti-inflammatory effects of the endogenous or exogenous glucocorticoids (GCs) by inhibiting their DNA binding [106]. Moreover, *in vivo* and *in vitro* studies have shown that, in addition to the reduction of anti-inflammatory GC effects, *β*-adrenergic agonists also stimulate eosinophil-mediated inflammation [107, 108]. Stress-induced *β*-AR stimulation also has proinflammatory effects on alveolar macrophages as demonstrated by enhanced secretion of IL-1*β* and TNF-*α*. This was completely stopped by *β*-adrenergic blockers but was increased even more by *α*-adrenergic blockade [109]. The findings of our study—significantly increased mRNA expression of proinflammatory cytokines and peribronchial foci of inflammation in lung histology—confirm a proinflammatory effect of *β*-adrenergic stimulation [40].

Some authors reported and discussed enhanced pulmonary capillary permeability after administration of *β*-AR agonists. This may result from an excessive increase in capillary pressure or from another primary cause of lung injury such as inflammation or endothelial damage. In an experimental model of oleic acid lung injury in dogs, terbutaline infusion aggravated capillary-alveolar leakage. The authors suggested that elevated cardiac output and reduced pulmonary vascular resistance would have increased perfusion surface by the recruitment of injured capillaries [110]. In our study with *β*-adrenergic infusion, pulmonary edema did not affect alveoli indicating that the capillary permeability was rather low [40, 50]. Induction of PE might be facilitated by desensitization and downregulation of *β*_2_-ARs after prolonged *β*-adrenergic stimulation. These effects were associated with a reduced increase in cAMP and in AFC [67, 111]. The impairment of AFC, however, occurred transiently after 48 h of infusion with ISO at a dose of 0.4 mg·kg^−1^·h^−1^ and then recovered after 96 h of continued infusion [112]. Moreover, the degree of AFC reduction was dose dependent: while moderate ISO doses (0.04 mg·kg^−1^·h^−1^) inhibited the terbutaline-induced AFC increase by 26%, AFC stimulation was not affected by low doses (0.004 mg·kg^−1^·h^−1^) of ISO [111]. In our study, we used continuous ISO infusion at the relatively low dose of 0.024 mgkg^−1^·h^−1^, and this transient impairment of AFC may account for edema formation that developed rather slowly [40, 50]. 

Taken together, despite numerous *β*-adrenergic effects that contribute to prevention and resolution of PE, these substances may also provoke formation of PE. The hemodynamic effects of *β*-adrenergic stimulation can increase pulmonary blood volume and, hence, fluid filtration. Moreover, inflammatory processes may be advanced, thus further enhancing fluid filtration. PE can result when the normal regulation of pulmonary fluid transport is overridden, for example, when compensatory mechanisms of the lung such as AFC or formation of pleural effusion are compromised or overcharged (see Figure 2(b)).

## 6. Conclusion

Endogenous and exogenous CAs can induce PE. Development of PE has been observed with *α*-adrenergic but also with *β*-adrenergic stimulation. The main mechanisms involved in PE formation are depicted in Figure 2. With *α*-adrenergic stimulation, PE mainly results from increased pulmonary arterial and capillary pressure due to strong generalized vasoconstriction. It can be maintained and aggravated by inflammation and disruption of the alveolar-capillary barrier. *β*-adrenergic stimulation increases cardiac output and leads to generalized vasodilation. This may ensue in pulmonary overperfusion and can cause PE when compensatory mechanisms are overridden.

## Abbreviations

AFC: Alveolar fluid clearance
ALI: Acute lung injury
AR: Adrenergic receptor
ARDS: Acute respiratory distress syndrome
BALF: Bronchoalveolar lavage fluid
CA: Catecholamine
GC: Glucocorticoid
HAPE: High-altitude pulmonary edema
i.v.: Intravenous
IL: Interleukin
ISO: Isoproterenol
LPS: Lipopolysaccharide
LV: Left ventricular
NE: Norepinephrine
NPE: Neurogenic pulmonary edema
PE: Pulmonary edema
PF: Pleural fluid
PHE: Phenylephrine
RVSP: Right ventricular systolic pressure
TNF: Tumor necrosis factor
TPR: Total peripheral resistance.
